# Electrical properties, phase transition, and conduction mechanism of the novel pyrophosphate compound Li_2_V_0.4_P_2_O_7_ compared with Li_2_Cu_(1−(5/2)*x*)_V_*x*_P_2_O_7_ (*x* = 0 and 0.2)

**DOI:** 10.1039/d6ra02485h

**Published:** 2026-07-03

**Authors:** Marwa Krichen, Makram Megdiche

**Affiliations:** a Laboratory LaSCOM, University of Sfax BP1171 3000 Sfax Tunisia krichen_marwa@hotmail.fr; b Laboratory of Composite Materials, Ceramics and Polymers, Physics Department, Science Faculty 3038 Sfax Tunisia

## Abstract

The present study investigates the effect of aliovalent substitution of Cu^2+^ by V^5+^ on the electrical and dielectric properties of the Li_2_Cu_(1−5/2)*x*_V_*x*_P_2_O_7_ series. Attention is mainly devoted to the synthesis and characterization of the fully substituted compound Li_2_V_0.4_P_2_O_7_ (*x* = 0.4), prepared by the ceramic method, and its behavior is compared to that previously reported for the compositions *x* = 0 and *x* = 0.2. Phase identification is confirmed by X-ray diffraction (XRD), revealing that the Li_2_V_0.4_P_2_O_7_ compound crystallizes in the monoclinic system with the *P*2_1_/*c* space group, while infrared spectroscopy (IR) confirms the formation of the P_2_O_7_ group, and differential thermal analysis (DTA) shows a phase transition at 640 K. Electrical and dielectric performances are investigated over wide temperature (574–713 K) and frequency (209 Hz to 5 MHz) ranges using complex impedance spectroscopy. The DC conductivity exhibits two thermally activated regions separated by the phase transition at 640 K. For the Li_2_V_0.4_P_2_O_7_ compound, the total conductivity reaches 6.15 × 10^−5^ Ω^−1^ cm^−1^ at 603 K (below the transition temperature) and 66.3 × 10^−5^ Ω^−1^ cm^−1^ at 683 K (above the transition temperature), confirming the thermally activated nature of charge transport from the analysis of the frequency exponent *s*. It follows that the conduction mechanism changes from non-small polaron tunneling (NSPT) below the transition temperature to correlated barrier hopping (CBH) at higher temperatures. A comparison among the three compositions indicates that aliovalent V^5+^ substitution leads to a systematic increase in ionic conductivity with increasing vanadium content, which may be attributed to the enhanced mobility of Li^+^ ions within the crystal lattice, thereby promoting charge transport. The sample with *x* = 0.4 exhibits the best electrical performance.

## Abstract 2

Progressive aliovalent substitution of Cu^2+^ by V^5+^ in Li_2_Cu_(1−(5/2)*x*)_ V_*x*_P_2_O_7_ leads to structural changes and improved ionic conduction.

## Graphical abstract

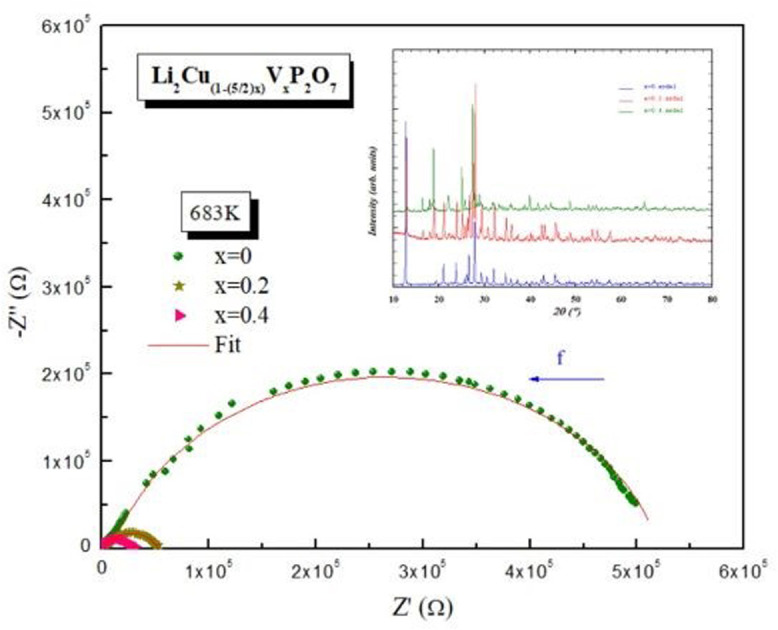

## 1 Introduction

Lithium ion-batteries (LIBs) dominate the current energy-storage technologies, setting increasingly severe requirements on the electrode material performance and stability.^1^ In this frame, pyrophosphates are a technologically important class of compounds owing to their rich physico-chemical properties, enabling applications in electronic devices, solid electrolytes with high thermal stability, sensors, semiconductor lasers, luminescent materials, ceramics, catalysts, ionic conductors, dielectrics, and magnetic or fluorescent systems.^2^ Lithium pyrophosphates of the type Li_2_MP_2_O_7_ (M = Ni, Zn, Cu, Ba, and Sr) belong to the broader A_2_BP_2_O_7_ family, characterized by the coexistence of an alkali cation (A^+^) and a divalent cation (B^2+^). Their structural diversity is outstanding, and these materials cannot be classified easily according to conventional criteria such as ionic size, coordination environment, or the type of bonding.^3–7^

There are several lithium pyrophosphates reported in the existing literature that possess a vast range of structural as well as electrical characteristics, depending on the nature of the metal ion present and the type of crystal formed. It has been previously established that any alteration of the metal ion can lead to structural changes, thereby affecting the transport of lithium ions.^8^

Among these pyrophosphates, the Li_2_CuP_2_O_7_ compound has already been studied and published,^9^ including its structural, vibrational, calorimetric, electrical, and dielectric properties. However, vanadium-substituted derivatives of this system remain less explored in the literature. In this context, an aliovalent substitution of the divalent Cu^2+^ cation by vanadium V^5+^ was performed to improve its electrical behavior. In a previously published work,^10^ the series of Li_2_Cu_(1−5/2)*x*_V_*x*_P_2_O_7_ (*x* = 0 and 0.2) samples were synthesized and characterized, and the results demonstrated that vanadium incorporation enhances ionic conduction across the entire studied temperature range. In particular, vanadium was chosen for its high chemical activity and its several oxidation states, which make it one of the most commonly used doping elements in functional materials.^11^

In addition, studies on lithium pyrophosphates containing vanadium have shown that the presence of vanadium may lead to structural modifications with a significant impact on lithium-ion diffusion properties, making it clear that aliovalent substitution is a powerful tool for controlling the physicochemical properties of lithium pyrophosphates.^12^

On the basis of previously obtained results, a higher substitution level (*x* = 0.4) was investigated to obtain the Li_2_V_0.4_P_2_O_7_ compound, with the aim of further enhancing ionic mobility and studying the effects of higher vanadium content on the structure and electrical properties. This composition can be considered the end member of complete substitution in the Li_2_Cu_(1−5/2)*x*_V_*x*_P_2_O_7_ series. It offers a comparative study with the other two compositions, *x* = 0 and *x* = 0.2, giving us more insight into the effect of increasing vanadium content in this series of compounds. This fully substituted composition has not yet been reported, and its structural and electrical properties remain unexplored. The present paper reports, for the first time, the synthesis and structural, vibrational, and calorimetric characterization of Li_2_V_0.4_P_2_O_7_, together with the study of its electrical properties and conduction mechanism over wide temperature and frequency ranges using complex impedance spectroscopy.

## 2 Experimental procedure

### 2.1 Synthesis procedure

The Li_2_V_0.4_P_2_O_7_ compound was synthesized *via* the conventional solid-state reaction method. High-purity (99%, Sigma Aldrich) analytical-grade precursors, including lithium carbonate (Li_2_CO_3_), vanadium pentoxide (V_2_O_5_), and ammonium dihydrogen phosphate (NH_4_H_2_PO_4_), were used. The starting materials were weighed in stoichiometric proportions according to the following reaction:
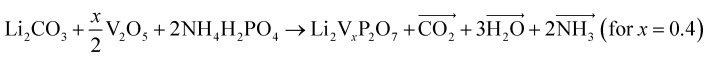


The powders were thoroughly mixed and ground using an agate mortar. The resulting homogeneous mixture was calcined at 573 K for 14 hours at a heating rate of 5 °C min^−1^ in an electric furnace to remove volatile components. After calcination, the powder was reground and pressed into pellets using a uniaxial pressure of 3 T cm^−2^ to facilitate the reaction. The pellets were then sintered at 813 K for 13 hours with a heating rate of 2 °C min^−1^ to obtain the final Li_2_V_0.4_P_2_O_7_ phase. After sintering, the furnace was allowed to cool naturally to room temperature.

The dissociation of V_2_O_5_ into V_2_O_4_ and O_2_ occurs only at temperatures above 973 K,^13^ as shown in the following reaction:

1 2V_2_O_5_ ↔ 2V_2_O_4_ + O_2_

Therefore, under our synthesis conditions, which involve temperatures below 973 K, vanadium is expected to remain in the +5 oxidation state.

### 2.2 Characterization

The synthesized compound was characterized to assess its phase purity and structural uniformity using X-ray powder diffraction. The measurements were carried out at ambient temperature within the angular interval of 4° < 2*θ* < 80°, employing a step increment of 0.02°, on a Philips PW 1710 diffractometer utilizing Cu Kα_1_ radiation (*λ* = 1.5406 Å). Structural analysis and refinement of the identified phases were performed through the Rietveld method, using a combination of FullProf, Celeref 2, and FOX software. For vibrational characterization, infrared spectra were recorded with a SPECTRUM BK spectrophotometer across the wavenumber range of 1300–500 cm^−1^.

A differential thermal analysis (DTA) measurement was carried out using a Linseis thermal analysis DTA-PT 1600 apparatus.

For electrical characterization, a pellet with a thickness of 1.1 mm and a diameter of 8 mm was prepared. Impedance measurements, including both real and imaginary components, were conducted over a broad temperature range (574–713 K) and frequency range (209 Hz to 5 MHz) using a TEGAM 3550 ALF automatic impedance analyzer, connected to a microcomputer and a compatible temperature controller. To ensure optimal electrical contact, both faces of the pellet were coated with a thin layer of gold and positioned between two copper electrodes within a dedicated sample holder. The impedance data were recorded while cooling from 713 K.

## 3 Results and discussion

### 3.1 X-ray analysis

**Fig. 1:**
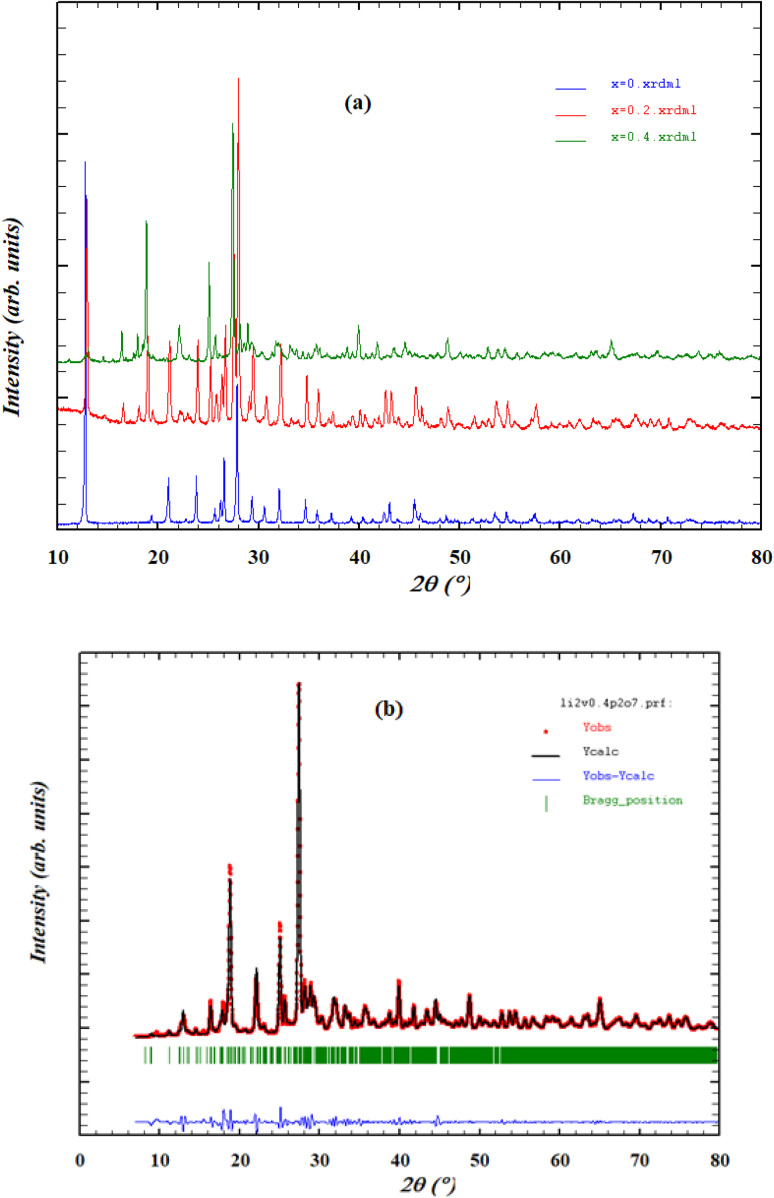
(a) X-ray diffractograms of Li_2_CuP_2_O_7_ (*x* = 0), Li_2_Cu_0.5_V_0.2_P_2_O_7_ (*x* = 0.2) and Li_2_V_0.4_P_2_O_7_ (*x* = 0.4) in the 2*θ* range 10°–80°. (b) Powder X-ray diffraction pattern and Rietveld refinement of the sample Li_2_V_0.4_P_2_O_7_ (circles correspond to the experimental data, and the calculated data are represented by the continuous line overlapping them; tick marks represent the positions of the allowed reflection, and a difference curve on the same scale is plotted at the bottom of the pattern).

Following our previous study^10^ that demonstrated the effective incorporation of vanadium into the Li_2_CuP_2_O_7_ framework for *x* = 0.2, the present work advances this investigation by increasing the vanadium concentration to *x* = 0.4. The synthesized sample Li_2_Cu_(1−5/2)*x*_V_*x*_P_2_O_7_ (*x* = 0.4) was characterized using powder X-ray diffraction. The diffraction patterns obtained at room temperature for compositions *x* = 0, 0.2, and 0.4 (Fig. 1(a)) clearly show structural modifications induced by vanadium substitution. In particular, the appearance of new diffraction peaks and the disappearance of others as *x* increases indicate a progressive rearrangement of the crystal structure. These spectral changes are associated with modifications in the diffraction profile and reflect significant alterations in the lattice symmetry and unit cell parameters due to the incorporation of vanadium into the structure.

Using several tools, including FullProf, CELREF, and FOX, Rietveld refinement of the powder XRD data was performed in order to better examine the structural characteristics of the prepared compound Li_2_V_0.4_P_2_O_7_. The structural models of comparable known compounds served as a reference for this refinement in order to guarantee precise fitting and structural interpretation.

A pseudo-Voigt function was used to model the peak profiles, while the background was estimated by linear interpolation between several points, whose heights were treated as adjustable parameters. The X-ray diffraction pattern of the synthesized Li_2_V_0.4_P_2_O_7_ compound is shown in Fig. 1(b). Rietveld refinement was performed to extract detailed structural information. The Li_2_V_0.4_P_2_O_7_ sample displays sharp and intense diffraction peaks, reflecting a high degree of crystallinity. All reflections are consistent with a monoclinic structure belonging to the *P*2_1_/*c* space group. After several iterative refinement steps and optimizations, all peaks were successfully indexed. The refined lattice parameters for the compositions Li_2_Cu_(1−5/2)*x*_V_*x*_P_2_O_7_ (*x* = 0, 0.2, and 0.4) are summarized in Table 1. Notably, the FullProf refinement did not reveal any secondary phases. The reliability factors—namely the profile *R*-factor (*R*_p_), the weighted profile *R*-factor (*R*_wp_), and the expected *R*-factor (*R*_exp_)—were all below 10%, confirming the high phase purity of the sample and the absence of significant impurities.

**Table 1:** Crystal data of the Li_2_Cu_(1−(5/2)*x*)_V_*x*_P_2_O_7_ (*x* = 0, 0.2 and 0.4) compound

Sample	Li_2_CuP_2_O_7_	Li_2_Cu_0.5_V_0.2_P_2_O_7_	Li_2_V_0.4_P_2_O_7_
*X*	0	0.2	0.4
Color	Blue	Green blue	Green
2*θ* (°) range	10–60	6–82	7–80
System	Mono	Mono	Mono
*a* (Å)	14.084(9)	15.083(1)	15.875(1)
*b* (Å)	4.871(3)	7.872(6)	6.506(5)
*c* (Å)	8.622(5)	12.329(8)	13.749(1)
*α*, *γ* (°)	90.00	90.00	90.00
*β* (°)	98.97(0)	91.092(3)	94.770(4)
Volume (Å^3^)	584.26(6)	1463.79(2)	1415.24(1)
Space group	*I*2/*a*	*P*2_1_/*n*	*P*2_1_/*c*
*Z*	8	4	4
*R*_p_ (%)	5.48	4.51	2.95
*R*_wp_ (%)	7.40	6.55	5.16
*R*_exp_ (%)	3.48	2.72	2.79
Bragg *R*-factor (%)	3.48	0.646	1.64
RF-factor (%)	4.06	0.318	1.12
*χ* ^2^	4.53	5.79	3.43

### 3.2 IR spectroscopic analysis

**Fig. 2:**
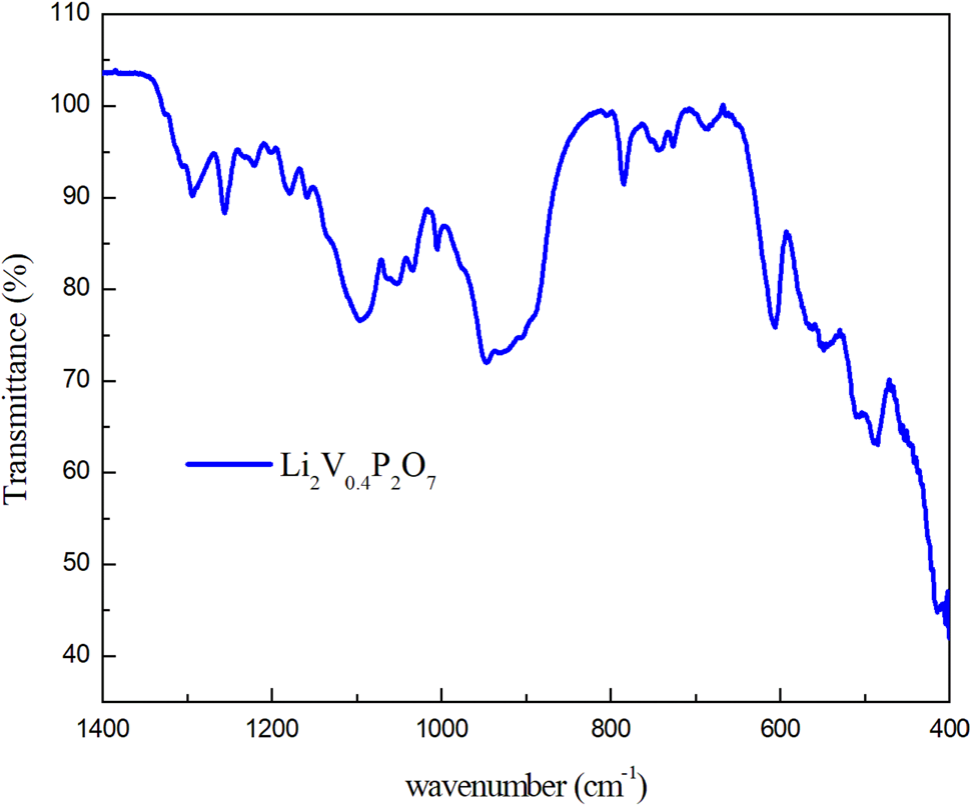
Infrared analysis spectrum of Li_2_V_0.4_P_2_O_7_ at room temperature.

The infrared spectrum of the Li_2_V_0.4_P_2_O_7_ compound, recorded at room temperature, is presented in Fig. 2 and displays transmittance as a function of wavenumber. By comparison with similar phosphate-based compounds, the most prominent bands have been attributed to specific vibrational modes. According to Corbridge,^14^ the P_2_O_7_^4−^ group, represented as O_3_P–O–PO_3_, can be interpreted as a combination of the vibrational modes of the PO_3_ units and the bridging P–O–P group. In addition to these characteristic modes, extra absorption bands are observed, which are attributed to VO_3_ group vibrations, indicating the incorporation of vanadium into the structure. These assignments, summarized in Table 2, confirm the presence of diphosphate groups in the title compound.

**Table 2:** Band assignment (cm^−1^) of Li_2_V_0.4_P_2_O_7_

Bands (cm^−1^) Li_2_V_0.4_P_2_O_7_	Assignment
1294	*ν*_as_(PO_3_)
1256	
1201	
1178	
1159	
1135	
1097	
1064	*ν*_s_(PO_3_)
1052	
1034	
1005	*ν*_as_(PO_3_)
904	*ν*_s_(VO_3_)
783	*ν*_as_(VO_3_)
948	*ν*_as_(P–O–P)
925	
755	*ν*_s_(P–O–P)
743	
726	
688	*ν*_as_(P–O–P)
605	*δ*(PO_3_) + *ρ*(PO_3_)
563	
508	
485	
414	

### 3.3 Calorimetric study

**Fig. 3:**
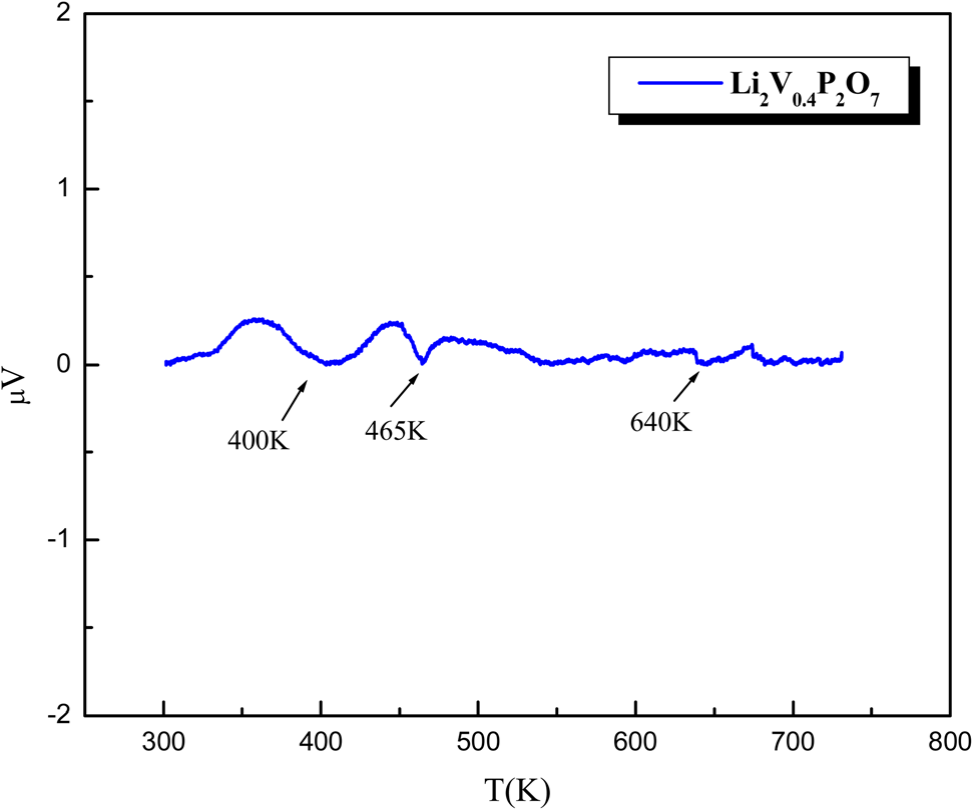
Thermogravimetric analysis of the Li_2_V_0.4_P_2_O_7_ compound.

The differential thermal analysis (DTA) curve shown in Fig. 3 exhibits three well-defined endothermic events that characterize the thermal behavior of the material. The first endothermic peak, appearing near 400 K, is most likely associated with the release of physically adsorbed or weakly bound water within the sample.^15^

Two additional endothermic peaks are observed at 465 K and 640 K, suggesting the presence of thermally activated structural transformations within the material.

To identify the nature of these transitions, the associated entropy changes were evaluated using Boltzmann's relation:

2 Δ*S* = *R* Ln *Ω* = *R* Ln(*N*_2_/*N*_1_)

where *Ω* = *N*_2_/*N*_1_, with *N*_1_ and *N*_2_ representing the numbers of accessible configurations before and after the phase transition, respectively. When the entropy change exceeds 2 J mol^−1^ K^−1^, the transition is typically classified as an order–disorder process. In contrast, lower entropy values (Δ*S* < 2 J mol^−1^ K^−1^) generally indicate a displacive-type mechanism.^16,17^

In our case, the entropy values obtained for the detected transition at 465 K and 640 K are Δ*S* = 0.299 J mol^−1^ K^−1^ (*Ω* = 1.036) and Δ*S* = 0.189 J mol^−1^ K^−1^ (*Ω* = 1.023), respectively, confirming that both transitions correspond to a displacive-type structural transition.

Both parent compounds, Li_2_Cu_(1−5/2)*x*_V_*x*_P_2_O_7_ (*x* = 0 and *x* = 0.2), have already been reported in the literature. When comparing our prepared sample to these references, we observe that the intermediate composition (*x* = 0.2) exhibits a significantly higher value of Δ*S*, as well as an increased value of *Ω*. Furthermore, the compound Li_2_V_0.4_P_2_O_7_ shows improved values compared to Li_2_CuP_2_O_7_.^9,10^

These results clearly indicate that vanadium substitution enhances the configurational disorder within the structure, especially for the intermediate composition Li_2_Cu_0.5_V_0.2_P_2_O_7_. This conclusion is supported by the marked increase in Δ*S* and by the corresponding rise in the configurational parameter *Ω*.

### 3.4 Electrical properties

The electrical properties were studied by complex impedance spectroscopy (CIS), which is a method that links theoretical circuit elements to the real electrical behavior of the material. CIS provides significant information on microstructural components such as grains, grain boundaries, and electrode interfaces. Furthermore, this method allows for the extraction of important electrical parameters from the complex impedance over a wide range of frequencies and temperatures.^18–20^

The complex impedance is expressed as:^21^

3 Z* = *Z*′ + *jZ*″

where *Z*′ and *Z*″ represent the real and imaginary components, respectively. This decomposition offers a detailed and accurate representation of the material's overall electrical response.

**Fig. 4:**
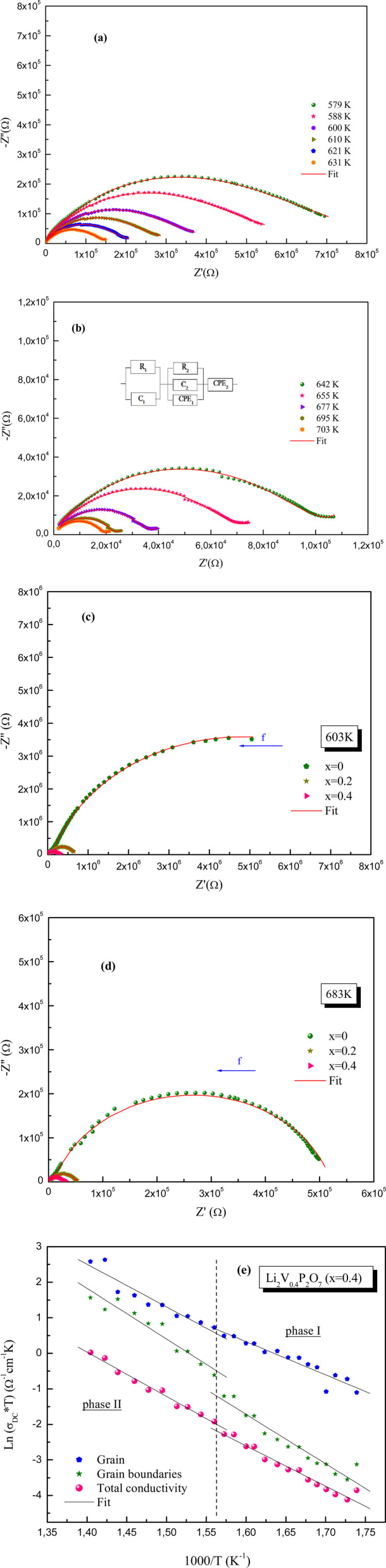
(a) Complex impedance spectra at low temperatures (phase I) and (b) high temperatures (phase II) of Li_2_V_0.4_P_2_O_7_ with (inset) the electrical equivalent circuit, complex impedance plots of Li_2_CuP_2_O_7_ (*x* = 0), Li_2_Cu_0.5_V_0.2_P_2_O_7_ (*x* = 0.2) and Li_2_V_0.4_P_2_O_7_ (*x* = 0.4) (c) at *T* = 603 K (phase I) and (d) at *T* = 683 K (phase II) and (e) variation of the Ln(*σT*) as a function of temperature for the Li_2_V_0.4_P_2_O_7_ sample.

Fig. 4(a) shows the Nyquist diagrams recorded at low temperatures, while Fig. 4(b) displays the corresponding spectra obtained at higher temperatures. The complex impedance plots exhibit two semicircular arcs, followed at very low frequencies by a short tail attributed to electrode polarization effects. For each temperature, the semicircles appear depressed below the real axis, indicating a distribution of relaxation times involved in the different conduction processes. This means that the electrical response does not follow the Debye model, but is consistent with the Cole–Cole formalism. In addition, the decreasing diameter of the semicircles with increasing temperature confirms the thermally activated nature of ionic conduction.

Using the ZView software, the experimental impedance data were fitted to the continuous lines shown in the figure. The obtained spectra were successfully modeled using a standard equivalent circuit presented in the inset of Fig. 4(b).^22^ This equivalent circuit consists of two cells connected in series. The first cell is composed of a resistance *R*_1_ in parallel with a capacitance *C*_1_, while the second cell includes a resistance *R*_2_ in parallel with a capacitance *C*_2_ and a constant phase element (CPE_1_). These two cells correspond to the bulk (grain) and grain boundary contributions, respectively. In addition, a fractal capacitance element (CPE_2_) is introduced in series to account for electrode polarization effects.

A constant phase element (CPE) is characterized by two parameters, *Q* and *α*, and its impedance is expressed as:^23^4
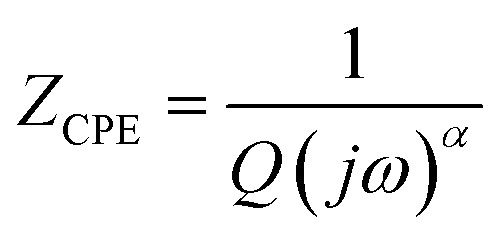
where *Q* has units of capacitance and *ω* is the angular frequency. The exponent *α* takes the values 1, 0, and 0.5 for ideal capacitive behavior, ideal resistive behavior, and Warburg-type diffusion, respectively.

Fig. 4(c and d) demonstrate a strong consistency between the experimental data (scatter) and the fitted theoretical curves (solid lines) for both the real and imaginary components of the impedance, confirming that the selected equivalent circuit provides an accurate representation of the electrical response of the material across the investigated temperature range.

It is also observed that the grain resistance (*R*_1_) is considerably lower than the grain boundary resistance (*R*_2_), indicating that ionic transport within the material is predominantly governed by grain boundary contributions. Moreover, the decrease of *R*_2_ with increasing temperature reflects the thermally activated nature of ionic conduction, likely dominated by Li^+^ mobility.^24^

**Fig. 5:**
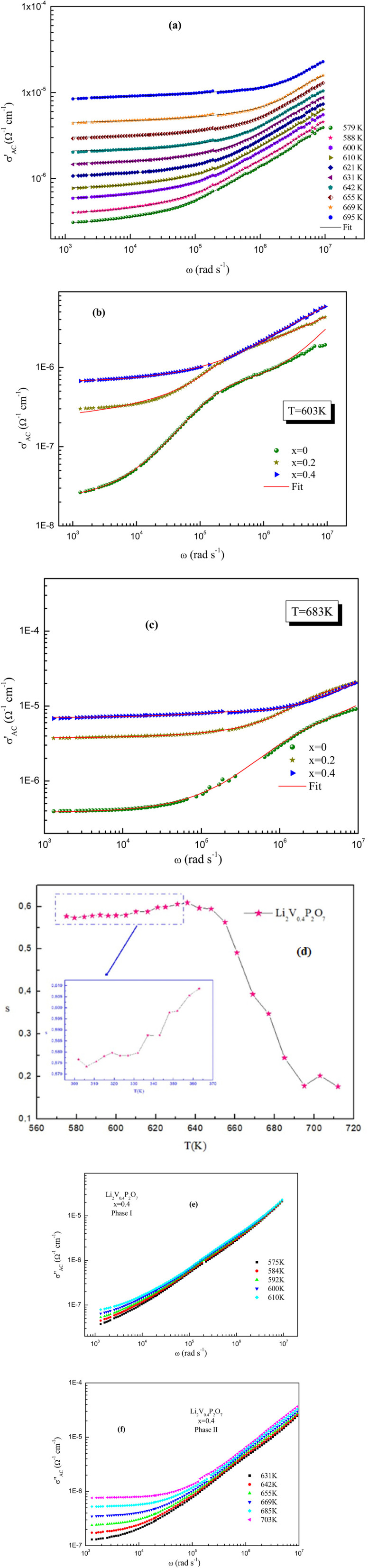
(a) Frequency dependence of the real part of the AC conductivity of Li_2_V_0.4_P_2_O_7_ at various temperatures, variation in the real part of the AC conductivity as a function of the angular frequency for Li_2_Cu_(1−(5/2)*x*)_V_*x*_P_2_O_7_ (*x* = 0, 0.2 and 0.4) (b) at *T* = 603 K (phase I) and (c) at *T* = 683 K (phase II), (d) variation of the universal exponent *s* as a function of temperature and frequency dependence of the imaginary part of the AC conductivity of Li_2_V_0.4_P_2_O_7_ (e) at low temperatures (phase I: *T* < 624 K) and (f) at high temperatures (phase II: *T* > 624 K).

To show the effect of substituting copper with vanadium, Fig. 5(c) and (d) present the Nyquist plots for the three compositions (*x* = 0, 0.2, and 0.4) at 603 K and 683 K. All samples display a similar overall impedance behavior. However, as the vanadium content increases, the radii of the semicircles become smaller. This decrease clearly indicates that both grain and grain boundary resistances are reduced when copper is replaced by vanadium. Therefore, vanadium substitution improves ionic transport in the material by lowering the resistive barriers to ion migration.

### 3.5 DC conductivity

The fitting of the Nyquist diagrams of the Li_2_V_0.4_P_2_O_7_ compound using the proposed equivalent circuit enables the extraction of the electrical parameters *R*_1_, *C*_1_, *R*_2_, *C*_2_, CPE_1_(*Q*), CPE_1_(*α*), CPE_2_(*Q*) and CPE_2_(*α*).

These parameters then allow the calculation of the dc conductivity (*σ*_DC_) of the grains, the grain boundaries, and the total conductivity at each temperature using the following expressions:^25^5
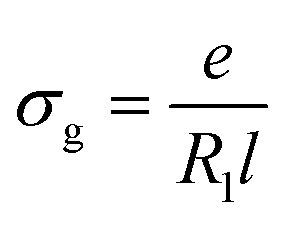
6
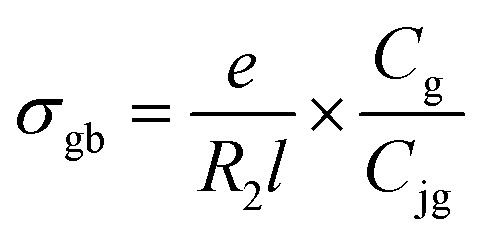
7
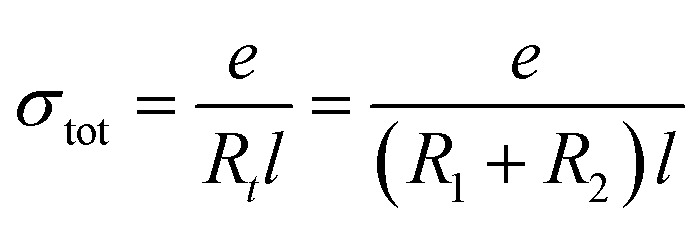
Where *e* and *l*, respectively, represent the thickness and the surface area of the sample-electrode contact. *C*_g_ and *C*_gb_ are the capacitances of the grains and grain boundaries, respectively, which are determined by the following equation:^26^

8 *C* = *R*^((1−*α*)/*α*)^*Q*^1/*α*^

The temperature evolution of the dc ionic conductivities corresponding to the various contributions is shown in Fig. 4(e). The curves display a linear variation with a noticeable change in slope around the transition temperature identified by the DSC analysis. Such linear behavior over the studied temperature range indicates that the conductivities obey an Arrhenius-type dependence:^27^9
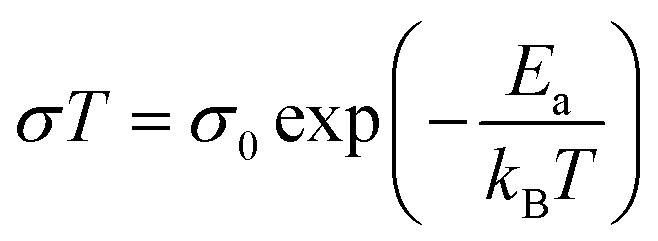
where *σ*_0_ is the pre-exponential factor, *E*_a_ is the activation energy for conduction, *T* is the absolute temperature, and *k*_B_ is the Boltzmann constant.

The activation energies and corresponding pre-exponential factors obtained from the Arrhenius fits are summarized in Table 3.

**Table 3:** Activation energies, pre-exponential factor and conductivities at 603 K (phase I) and 683 K (phase II) of grain, grain boundaries, and total conductivity for Li_2_V_0.4_P_2_O_7_

Li_2_V_0.4_P_2_O_7_ (*x* = 0.4)		Phase I	Phase II
Activation energy (eV)	Grain	0.81	1.04
	Grain boundary	1.20	1.23
	Total conductivity	0.98	1.08
Pre-exponential factor *σ*_0_ (Ω^−1^ cm^−1^ K)	Grain	2.47 × 10^6^	2.47 × 10^8^
	Grain boundaries	8.27 × 10^8^	2.95 × 10^9^
	Total conductivity	5.61 × 10^6^	3.94 × 10^7^
Conductivity (*T* = 603 K) (Ω^−1^ cm^−1^)	Grain	1.30 × 10^−3^	
	Grain boundary	12.77 × 10^−5^	
	Total conductivity	6.15 × 10^−5^	
Conductivity (*T* = 683 K) (Ω^−1^ cm^−1^)	Grain	8.11 × 10^−3^	
	Grain boundary	354 × 10^−5^	
	Total conductivity	66.3 × 10^−5^	

The conductivity at two selected temperatures—one in phase I (603 K) and another in phase II (683 K)—was calculated and reported in Table 3. By comparing these values with those previously published for the compounds Li_2_CuP_2_O_7_ and Li_2_Cu_0.5_V_0.2_P_2_O_7_,^9,10^ it is observed that increasing the vanadium content leads to an enhancement of the ionic conductivity.

This improvement may be associated with the small size of V^5+^, which allows better incorporation into the host lattice and facilitates ion mobility. The aliovalent V^5+^ substitution likely increases the concentration of mobile charge carriers through the formation of defect states, thereby enhancing ionic transport. Moreover, the conductivity increases with temperature (*σ*_DC_ (phase I) < *σ*_DC_ (phase II)) since thermal activation provides energy to ionize the defects and assist the migration of Li^+^ ions between the available lattice sites.

In order to gain additional insight into how the results obtained in the present study compare with those reported for lithium pyrophosphates, Table 4 summarizes the literature data for conductivity, activation energy, temperature range, and mechanism of conduction for a number of different Li_2_MP_2_O_7_ compounds. From this comparative analysis, it is clear that the cation of the metal plays a prominent role in determining the conduction characteristics of lithium pyrophosphates.

**Table 4:** Electrical conductivity of the Li_2_MP_2_O_7_-type compounds

Compound	Sys	Temperature range (K)	*E*_a_ (eV)	Conductivity (Ω^−1^ cm^−1^)		Conduction mechanism	Ref.
Li_2_BaP_2_O_7_	Mono	589–724	1.08	*σ*_675K_ = 3.05 × 10^−7^	*σ*_683K_ = 2.13 × 10^−6^	OLPT	4
Li_2_SrP_2_O_7_	Ortho	519–628	1.03	—	—	CBH	5
Li_2_CaP_2_O_7_	Tri	373–673	0.49	—	—	—	7
Li_2_CuP_2_O_7_	Mono	576–710	1.44	*σ*_603K_ = 0.213 × 10^−5^	*σ*_683K_ = 3.49 × 10^−5^	CBH (*T* < 622 K)	9
			1.26			OLPT (*T* > 622 K)	
Li_2_Cu_0.5_V_0.2_P_2_O_7_	Mono	574–713	0.87	*σ*_603K_ = 3.19 × 10^−5^	*σ*_683K_ = 38.9 × 10^−5^	NSPT (*T* < 624 K)	10
			1.19			OLPT (*T* > 624 K)	

### 3.6 AC conductivity and conduction mechanism

AC conductivity studies help to understand the conduction mechanism in the studied compound over the explored temperature and frequency range. Impedance spectroscopy measurements were used to determine the AC conductivity values. The values are calculated using impedance data through the following relation:^28^10
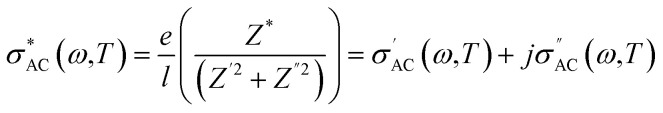


The real and the imaginary parts of the total AC conductivity, 
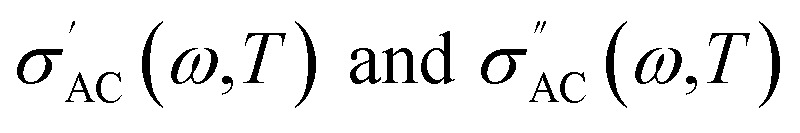
 are calculated using the following expressions:11
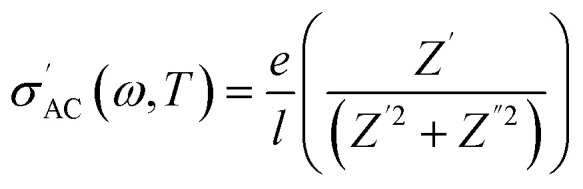
12
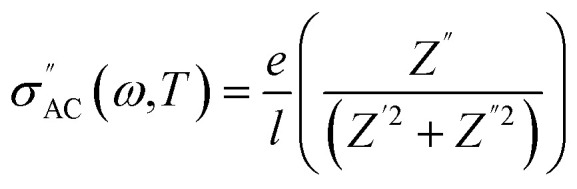
where *e*, *l*, *Z*′, and *Z*″, respectively, are the thickness, the cross-sectional area, and the real and the imaginary parts of complex impedance.

The frequency dependence of the real part of the AC conductivity, 
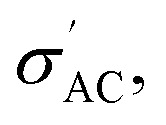
 at different temperatures is shown in Fig. 5(a). At low frequencies, the curves exhibit an almost frequency-independent plateau corresponding to the dc conductivity. This plateau reflects the long-range transport of charge carriers through hopping mechanisms. At higher frequencies, a dispersive region appears, which is attributed to complex interactions between mobile charge carriers and the lattice. This overall behavior is consistent with Jonscher's universal power law:^29^13
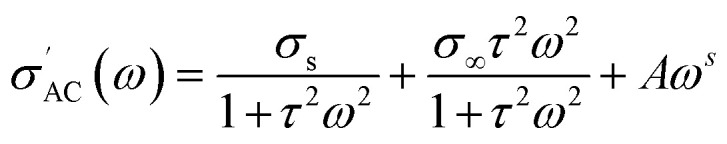
where *σ*_s_ is the conductivity at low frequencies, *σ*_∞_ is an estimate of the conductivity at high frequencies, *ω* = 2π*f* is the angular frequency, *τ* represents the characteristic relaxation time, *A* is a constant that is temperature-dependent, and *s* is the power law exponent, where 0 < *s* < 1.

The fitting of the experimental data, using Jonscher's equation, shows excellent agreement between the experimental points (scatter) and the theoretical fits (solid lines). This good agreement confirms that Jonscher's model accurately describes the AC conduction behavior in the compound under investigation.

Fig. 5(b) presents the evolution of 
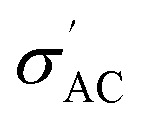
 for the different compositions Li_2_Cu_(1−5/2)*x*_V_*x*_P_2_O_7_ (*x* = 0, 0.2, and 0.4) at *T* = 683 K (phase I), while Fig. 5(c) shows the corresponding behavior at *T* = 603 K (phase II). The obtained curves reveal that increasing the vanadium content leads to a noticeable enlargement of the low-frequency plateau associated with *σ*_DC_, reflecting an improvement in the long-range charge transport. Also, this plateau is shifted upward for higher values of *x*, which implies an evident enhancement of the dc conductivity. Such an increase in conductivity can be explained by the increase in the mobile charge carriers' concentration and, correspondingly, in their mobility, facilitated by the structural and defect transformations caused by V^5+^ incorporation. In this context, the improvement in conductivity may be associated with enhanced Li^+^ mobility within the lattice.

The simulation of the 
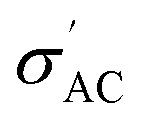
 slope by eqn (13) for the Li_2_V_0.4_P_2_O_7_ compound at different temperatures enables the extraction of the frequency exponent *s*. Its thermal evolution, shown in Fig. 5(d), makes it possible to determine the most probable conduction mechanism in the material and to follow its changes as the temperature increases.

According to the literature, there are different models explaining the behavior of this exponent, such as overlapping large-polaron tunneling (OLPT),^30^ non-overlapping small polaron tunneling (NSPT),^31^ quantum mechanical tunneling (QMT)^32^ and correlated barrier hopping (CBH).^33^

The variation of *s* reflects changes in the conduction mechanism. In phase I, *T* < 640 K, the *s* values increase with increasing temperature (note that the inset of Fig. 5(d) provides an enlarged view of this region), indicating that the non-overlapping small polaron tunneling (NSPT) model is consistent with the observed behavior. In phase II, *T* > 640 K, *s* decreases as the temperature increases, which suggests that the correlated barrier hopping (CBH) model governs the conduction process.

The frequency dependence of the imaginary part of the total AC conductivity, 
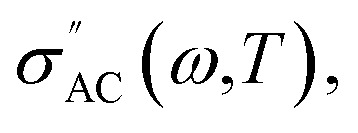
 obtained from eqn (12) for some temperatures below and above 640 K, is presented in Fig. 5(e) and (f), respectively. In the high-frequency region, during phase I (*T* < 640 K), an almost linear variation is observed, which remains independent of the temperature. The corresponding slope:^34^14
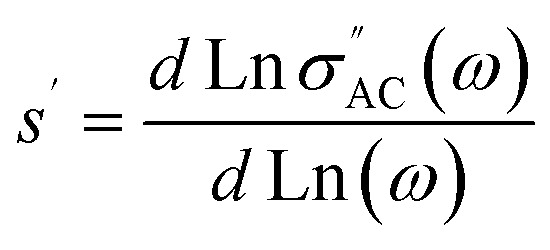
stays close to unity, indicating that dipolar reorientation mechanisms dominate the dielectric response in this regime.

In phase II (*T* > 640 K), a deviation from linearity appears in the low-frequency region, where 
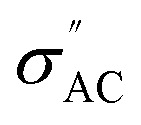
 increases markedly with temperature. This behavior reflects the increasing contribution of thermally activated charge carriers, which may be associated with ionic species such as Li^+^.

For the Li_2_V_0.4_P_2_O_7_ compound, a characteristic dispersive behavior is observed in the intermediate-frequency range: while the curves remain linear at high frequencies, they become increasingly dispersed toward the low-frequency side in phase II. This trend contrasts with that observed for the copper-based compounds Li_2_CuP_2_O_7_ and Li_2_Cu_0.5_V_0.2_P_2_O_7_, which exhibit dispersion extending across both low- and high-frequency regions.

This difference originates from the combined effects of structural symmetry and cation substitution. Li_2_CuP_2_O_7_ crystallizes in the *I*2/*a* space group, Li_2_Cu_0.5_V_0.2_P_2_O_7_ in *P*2_1_/*n*, whereas Li_2_V_0.4_P_2_O_7_ adopts the *P*2_1_/*c* symmetry. Such changes in the space-group symmetry modify the connectivity of the VO_6_/PO_4_ polyhedral and alter the local potential landscape experienced by charge carriers, thereby influencing their relaxation dynamics. Moreover, replacing Cu^2+^ (Jahn–Teller active, 3d^9^) with V^5+^ (non-degenerate, 3d^0^) reduces local distortions and can contribute to improved ionic mobility. This accounts for both the more regular high-frequency behavior and the distinct low-frequency dispersion observed in the vanadium-rich compound.

#### 3.6.1 The NSPT model (phase I)

In order to interpret the behavior of the frequency exponent *s* in region I for Li_2_V_0.4_P_2_O_7_, one notices that *s* remains below unity and increases progressively with temperature. Such a trend is characteristic of the Non-Overlapping Small Polaron Tunneling (NSPT) model, which therefore provides the most suitable description of the conduction mechanism in this regime.

In this model, the AC conductivity is given by:^35^15
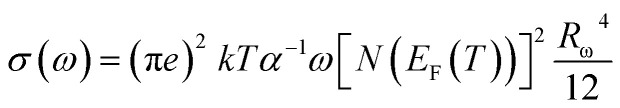
with16
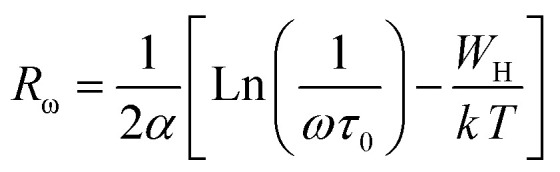


From the above equations, *α*^−1^ is the spatial extension of the polaron (=1 × 10^10^ m), *N* (*E*_F_) is the density of the states at the Fermi level, *R*_ω_ is the tunneling distance and *W*_H_ represents the polaron hopping energy.

Within the tunneling model, the frequency exponent *s* is given by:17
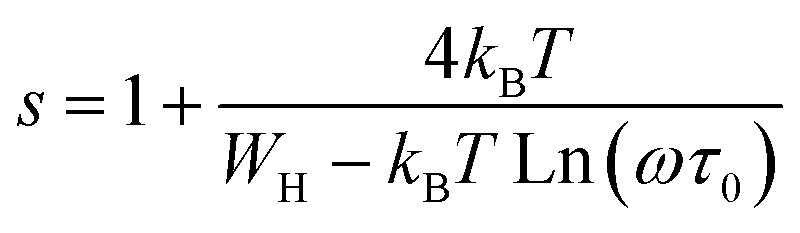
where *W*_H_ is the polaron hopping energy, *k*_B_ is the Boltzmann constant, and *τ*_0_ is the characteristic relaxation time, typically on the order of 10^−13^ s.

For large values of *W*_H_/*k*_B_*T*, *s* simplifies to18
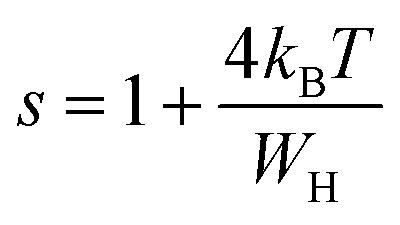


These relations highlight how NSPT conduction arises from thermally assisted tunneling of small polarons between localized states, with the exponent *s* capturing the sensitivity of the process to both temperature and frequency.

**Fig. 6:**
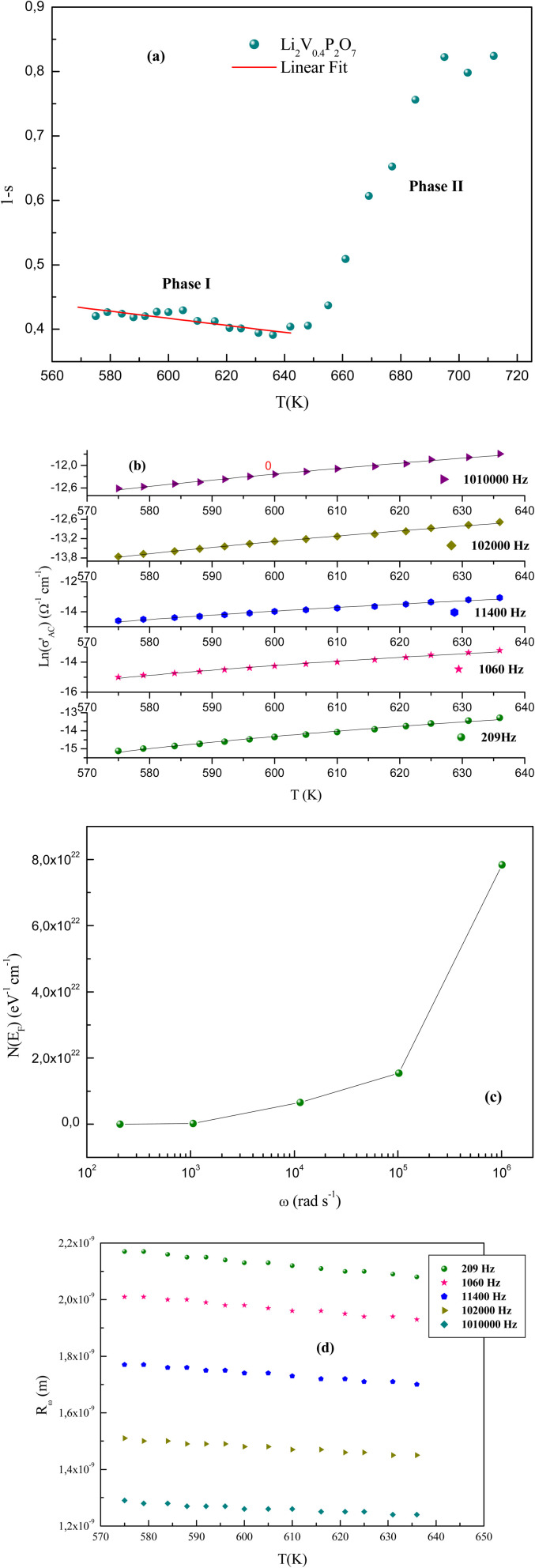
(a) Variation of 1 − *s* as a function of temperature, (b) fitting of the real part of the AC conductivity at different frequencies using the NSPT model, (c) variation of the parameter *N* (cm^−3^) as a function of frequency in region I and (d) temperature dependence of the tunneling distance *R*_ω_ (Å) at different frequencies for Li_2_V_0.4_P_2_O_7_.

The value of *W*_H_ was obtained from the slope of the (1 − *s*) *versus* temperature plot, leading to an activation energy of approximately 0.63 eV for Li_2_V_0.4_P_2_O_7_ (*x* = 0.4) (Fig. 6(a)).

The fitting of the 
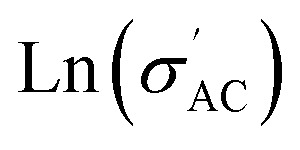
*versus* temperature curves using eqn (15) at different frequencies exhibits excellent agreement between the experimental data and the theoretical predictions (Fig. 6(b)), further confirming that the non-overlapping small polaron tunneling model accurately describes the conduction in phase I.

Moreover, the modeling allows the extraction of the key physical parameters-namely, the density of localized states *N*(*E*_F_) and the tunneling distance *R*_ω_. As shown in Fig. 6(c), *N*(*E*_F_) increases with increasing frequency, reflecting the greater availability of localized states participating in tunneling. In contrast, *R*_ω_ decreases with both temperature and frequency (Fig. 6(d)), indicating that charge carriers require shorter hopping distances as thermal activation and frequency rise. The obtained values of *R*_ω_ fall within the range of 1.2–2.2 Å, which is fully consistent with short-range polaron hopping.

#### 3.6.2 The CBH model (phase II)

According to the CBH model, conduction occurs through successive hops over the potential barrier separating two nearby defect centers. This model proposes that the AC conductivity arises from either single-polaron or bipolaron hopping across the Coulomb barrier between adjacent localized sites. The AC conductivity is influenced by several factors, including:^36^19
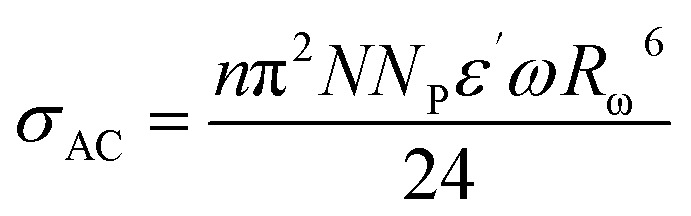
where *n* is the number of polarons involved in the hopping process (*n* = 1 and 2 for the single- and double–polaron processes, respectively), *NN*_p_ is proportional to the square of the density of states, *ε*′ is the dielectric constant, and *R*_ω_ is the hopping distance of the charge carriers responsible for conduction.

The hopping distance, defined by *R*_ω_ for a given frequency and temperature within the framework of the CBH model, is given by:^37^20
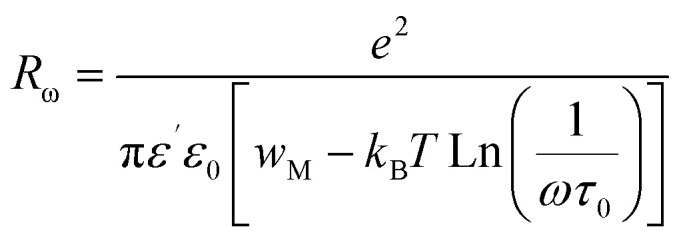
where *e* is the electron charge, *ε*_0_ is the vacuum permittivity, *k*_B_ is the Boltzmann constant, *T* is the temperature, and *τ*_0_ is the characteristic relaxation time.

In the bipolaron hopping process, the parameter *NN*_p_ can be related to the density of localized states *N*_T_ through:*NN*_p_ = *N*_T_^2^In the case of single-polaron hopping, the expression is:21
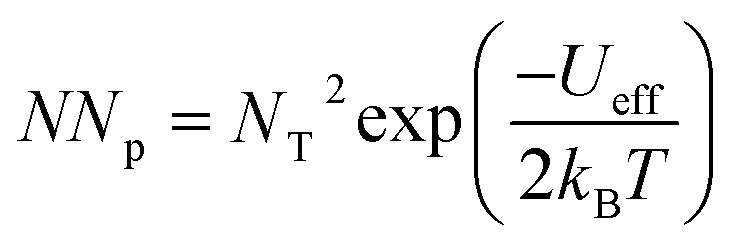
where *U*_eff_ is the effective correlation energy due to the electron–phonon interactions, which might become negative when the electron–phonon coupling is strong enough.

**Fig. 7:**
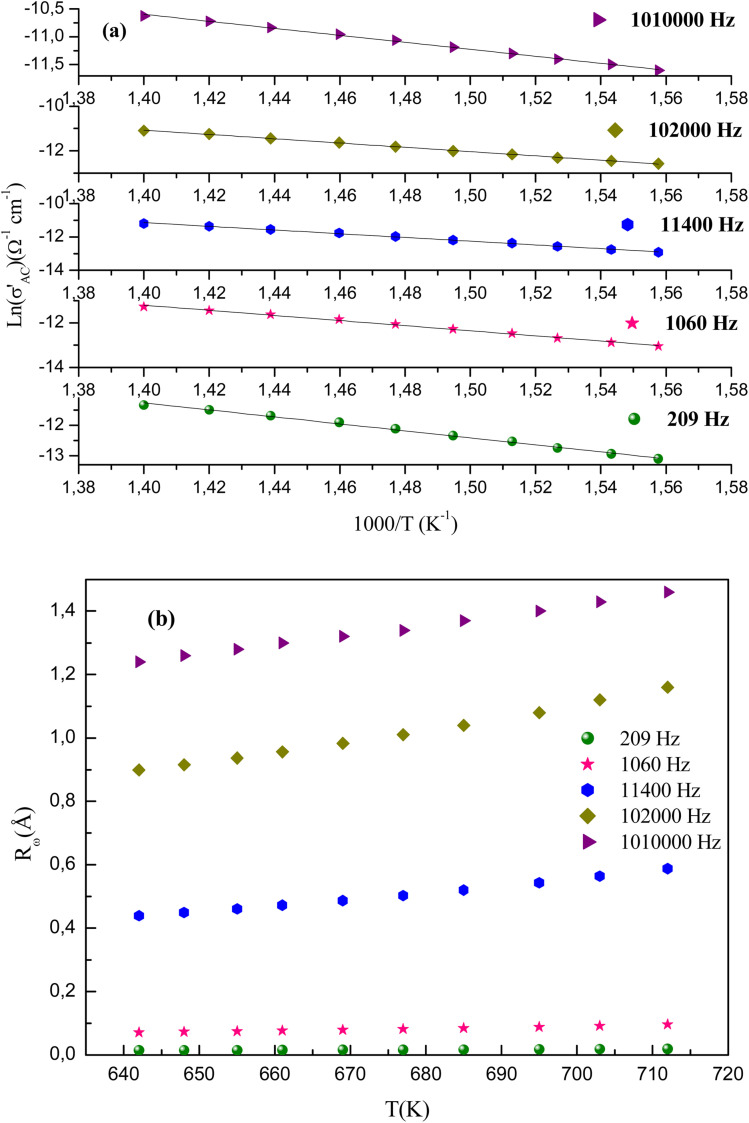
(a) Fitting of the real part of the AC conductivity at different frequencies using the CBH model and (b) temperature dependence of the tunneling distance *R*_ω_ (Å) at different frequencies for Li_2_V_0.4_P_2_O_7_.

Fig. 7(a) depicts the evolution of Ln *σ*_ac_ as a function of the inverse absolute temperature at various applied frequencies. The presented curves are characterized by a linear increase with a rise in temperature, which confirms that the AC conductivity follows the thermally activated mechanism that involves localized charge carriers. The lines in the figure are the best fits of the experimental data. This consequently denotes that AC conduction in lithium-vanadate pyrophosphate is mainly controlled by a bipolaron hopping process.

The different parameter values used in the fitting procedure are summarized in Table 5. We can conclude that the density of localized states decreases with an increase in frequency. Following Mott's explanation, such a decrease in the number of localized states with an increase in frequency can thus be considered to be due to an increase in the structural disorder, which reduces the stability of the localized states and favors their partial delocalization.^38^

**Table 5:** Experimental parameters extracted for different frequencies for the CBH model

Frequency (Hz)	*N* (eV^−1^ m^−1^)	*W*_m_ (eV)
209	6.48 × 10^25^	1.79
1060	5.98 × 10^23^	1.67
11 400	2.44 × 10^21^	1.49
102 000	1.35 × 10^20^	1.37
1 010 000	1.06 × 10^19^	1.36

The frequency variation of the hopping distance (*R*_ω_) for the Li_2_V_0.4_P_2_O_7_ compound is presented in Fig. 7(b). It is observed that *R*_ω_ increases monotonically with increasing frequency. This behavior is consistent with a decrease in the density of localized states (*N*), as shown in Table 5. Moreover, the potential barrier height *W*_m_ shows a slight decrease with frequency. This suggests that the energy barrier separating neighboring sites becomes lower at higher frequencies, meaning that charge carriers require less energy to perform short-range hopping. Consequently, the conduction process becomes increasingly localized as the frequency increases.

### 3.7 Thermodynamics constants

The relaxation time may be represented as follows using Eyring's theory:^39^22
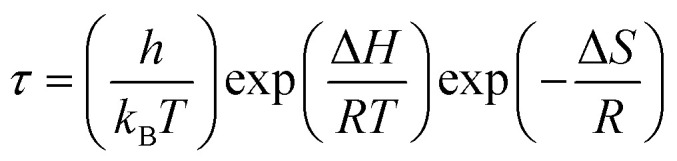
where *h* is Planck's constant, *R* is the universal gas constant, Δ*S* represents the migration entropy, and Δ*H* is the enthalpy of the system.

**Fig. 8:**
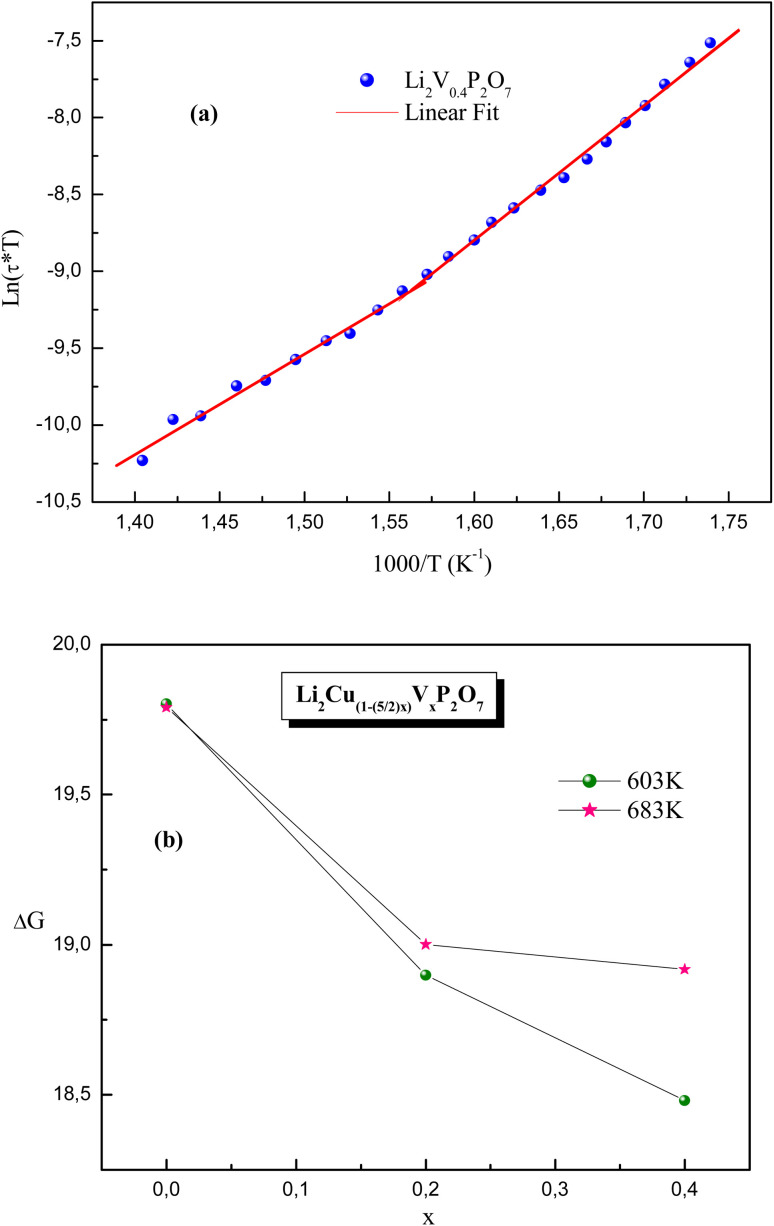
(a) Temperature dependence of Ln(*τT*) and (b) variation in Δ*G* as a function of Li_2_Cu_(1−(5/2)*x*)_V_*x*_P_2_O_7_ (*x* = 0, 0.2 and 0.4) at *T* = 603 K (phase I) and *T* = 683 K (phase II).

The thermodynamic parameters Δ*H* and Δ*S* can be extracted from the linear plot of Ln(τ*T) as a function of the inverse of temperature (Fig. 8(a)).

The curve shows Arrhenius-type behavior, and the slope clearly changes near the transition temperature that was identified by thermal analysis. This deviation allows the determination of the thermodynamic parameters separately for the two regions.

During phase I, the change in enthalpy of activation Δ*H* was determined to be 17.33 kcal mol^−1^, while the corresponding entropy (Δ*S*/*R*) was −0.959. The change in enthalpy Δ*H* is 12.721 kcal mol^−1^ during phase II, and entropy (Δ*S*/*R*) is −4.582.

Instead of reporting Δ*G* at single temperatures, the Gibbs free activation energy was calculated as a function of the vanadium substitution level using the following relation:

23 Δ*G* = Δ*H* − *T*Δ*S*

Fig. 8(b) shows the evolution of Δ*G* as a function of the vanadium content *x*, as computed at the characteristic temperatures of each phase, 603 K for phase I and 683 K for phase II.

A clear decreasing trend is observed: Δ*G* decreases with increasing vanadium content. This reduction in the activation free energy suggests that V^5+^ incorporation lowers the energy barrier for charge transport, which may be associated with enhanced Li^+^ mobility within the structure.

It should be noted that Δ*S* values are not necessarily positive. Negative values of Δ*S* correspond in general to dipole–dipole interactions, which indicate that the molecular entities are more ordered or more closely packed in the activated state.

## 4 Conclusion

The Li_2_V_0.4_P_2_O_7_ compound was prepared by the conventional ceramic method. X-ray diffraction analysis confirmed the formation of a single phase compound crystallizing in the monoclinic system with space group *P*2_1_/*c*. The replacement of Cu^2+^ by V^5+^ induces a significant structural evolution as compared to the end compositions (*x* = 0 and *x* = 0.2). Such an evolution is attested by the variation of the unit cell parameters and symmetry. IR spectroscopy confirms the presence of characteristic entities of the P_2_O_7_^4−^ anionic group, while the examination of the thermogram reveals a thermal transition at 640 K.

Impedance spectroscopy reveals thermally activated charge transport and the systematic enhancement of electrical conductivity with an increase in the vanadium content. An equivalent circuit was used to model the Nyquist plots, which allowed the refinement of parameters and the extraction of the DC conductivity at any specific temperature, *σ*_DC_. The thermal variation of *σ*_DC_ follows an Arrhenius-type dependence, although it shows a noticeable deviation around the transition temperature identified by thermal analysis. In addition, the thermal variation of the frequency exponent *s* reflects a variation in the predominant conduction mechanism: NSPT below the transition temperature gives way to a CBH at higher temperatures.

Overall, the comparison in the three studied compositions shows that aliovalent V^5+^ substitution significantly enhances charge mobility. This improvement may be associated with lattice distortions and increased structural disorder induced by the Cu^2+^ → V^5+^ substitution, which facilitates hopping transport within the pyrophosphate framework and may contribute to Li^+^ mobility. The *x* = 0.4 composition thus presents the best electrical performance within this series. These results highlight the potential of this material for solid-state functional applications involving thermally activated charge transport.

## Conflicts of interest

There are no conflicts to declare.

## Data Availability

The data that support the findings of this study are available from the corresponding author upon reasonable request.

## References

[cit1] Li, Zhu Z., Hu S., Wu Y., Wan C., Gao R., Yao H., Lan H., Liu Z., Wang Z., Li H. (2025). Amorphous tin pyrophosphate enabled by inositol hexaphosphate chelation for ultrahigh-capacity lithium-ion battery anodes. Chem. Eng. J..

[cit2] Mortadi H., Bouragba F. Z., Assekouri A., Alaoui belghiti H. E. L., Sabbar E., Bettach M. (2022). Investigation of optical, electrical and dielectric properties of pyrophosphates Li_x_Cu_2-x/2_ P_2_O_7_ (x = 0.0; 0.05; 0.1). J. Solid State Chem..

[cit3] Karray M., Nasri S., Mendil R., Kammoun I., Mahmoud A., Oueslati A. (2023). Studies on Structural and Ionic Conductivity of Li_2_NiP_2_O_7_. ECS J. Solid State Sci. Technol..

[cit4] Krichen M., Megdiche M., Guidara K., Mohamed G. (2015). AC conductivity and mechanism of conduction study of lithium barium pyrophosphate Li_2_BaP_2_O_7_ using impedance spectroscopy. Ionics.

[cit5] Ajili O., Louati B., Guidara K. (2018). Dielectric and ac ionic conductivity investigation of Li_2_SrP_2_O_7_. Indian J. Phys..

[cit6] Hamdi M., Shuheil M. A., Oueslati A. (2024). Synthesis, morphological, and ionic conductivity of a lithium cerium diphosphate compound. Ionics.

[cit7] Madkhali O. (2026). Li_2_CaP_2_O_7_ pyrophosphate ceramics with dual functionality: high-performance NTC thermistor behavior and giant dielectric permittivity. RSC Adv..

[cit8] Smiley D. L., Tessaro M. Z., He X., Goward G. R. (2015). Correlation of Electrochemical Performance with Lithium Environments and Cation Dynamics in Li_2_(Mn_1-y_Fe_y_) P_2_O_7_ using ^6^Li Solid-State NMR. J. Phys. Chem. C.

[cit9] Krichen M., Mohamed G., Guidara K., Megdiche M. (2017). Phase transition and electrical investigation in lithium copper pyrophosphate compound Li_2_CuP_2_O_7_ using impedance spectroscopy. Ionics.

[cit10] Marwa K., Makram M. (2024). Structural, vibrational, electrical and dielectric investigation of pyrophosphate compounds Li_2_Cu_(1-(5/2)x)_ V_x_P_2_O_7_ (x = 0 and 0.2). J. Alloys Compd..

[cit11] Rousse, Wurm C., Morcrette M., Rodriguez-Carvajal J., Gaubicher J., Masquelier C. (2001). Crystal structure of a new vanadium(IV) diphosphate: VP_2_O_7_, prepared by lithium extraction from LiVP_2_O_7_. Int. J. Inorg. Mater..

[cit12] Xu J., Chou S.-L., Gu Q.-F., Md Din M. F., Liu H.-K., Dou S.-X. (2014). Study on Vanadium Substitution to Iron in Li_2_FeP_2_O_7_ as Cathode Material for Lithium-ion Batteries. Electrochim. Acta.

[cit13] Jon Hawes Clark R. , The Chemistry of Titanium and Vanadium, Elsevier, Amsterdam, The Netherlands, 1968

[cit14] D E Corbridge Topics in Phosphorus Chemistry, ed. E. J. Griflith, New York, J Wiley, p. 57, 1966

[cit15] Szklarz P., Owczarek M., Bator G., Lis T., Gatner K., Jakubas R. (2009). Crystal structure, properties and phase transitions of morpholinium tetrafluoroborate [C_4_H_10_NO][BF_4_]. J. Mol. Struct..

[cit16] Czupin´ski O., Wojtas M., Pietraszko A., Jakubas R. (2007). Structural characterization, phase transition and dielectric properties of 4-cyanopyridynium perchlorate monohydrate: [(4-CNC_5_H_4_NH)][ClO_4_]·H_2_O. Solid State Sci..

[cit17] Karoui K., Ben Rhaiem A., Jomni F., Moneger J. L., Bulou A., Guidara K. (2013). Characterization of phase transitions of [N(CH_3_)_4_]_2_ZnCl_2_Br_2_ mixed crystals. J. Mol. Struct..

[cit18] Elferjani A., Abdelhedi M., Dammak M. (2016). *et al.*, Structural, dielectric and vibrational studies of the new mixed solid solution of thallium potassium sulfate selenate tellurate. Appl. Phys. A.

[cit19] Panda D., Hota S. S., Choudhary R. N. P. (2023). Investigation of structural, microstructural, dielectric, and electrical characteristics of a new lead-free compound: Ca_3_Bi_2_MoO_9_. J. Mater. Sci.: Mater. Electron..

[cit20] Sekhar Hota S., Panda D., Choudhary R. N. P. (2023). Studies of structural, dielectric, and electrical properties of polycrystalline barium bismuth tungstate for thermistor application. Inorg. Chem. Commun..

[cit21] Essaleh L., Marín G., Wasim S. M., Lahlali S., Chehouani H. (2016). Analysis of complex impedance of p-CuIn_3_Se_5_ by impedance spectroscopy. J. Alloys Compd..

[cit22] ben Abdessalem M., Aydi A., Abdelmoula N. (2019). Raman scattering, structural, electrical studies and conduction mechanism of Ba_0.9_Ca_0.1_Ti_0.95_Zr_0.05_O_3_ ceramic. J. Alloys Compd..

[cit23] Ben Yahya S., Garoui I., Zaghrioui M., Oueslati A., Louati B. (2025). Solid-state synthesized Li4GeO4 germanate: an exploration of its structure, vibrational characteristics, electrical conductivity, and dielectric properties. RSC Adv..

[cit24] Ben Bechir M., Akerm M. (2024). Structural, morphological, electrical, and dielectric properties of Na2Cu5(Si2O7)2 for ASSIBs. RSC Adv..

[cit25] Enneffatia M., Rasheed M., Louatia B., Guidaraa K., Shihab S., Barillé R. (2021). Investigation of structural, morphology, optical properties and electrical transport conduction of Li0.25Na0.75CdVO4 compound. J. Phys.: Conf. Ser..

[cit26] Chinarro E., Jurado J. R., Figueiredo F. M., Frade J. R. (2003). Bulk and grain boundary conductivity of Ca0.97 Ti1− xFexO3− δ materials. Solid State Ionics.

[cit27] Khiar A. S. A., Puteh R., Arof A. K. (2006). Conductivity studies of a chitosan-based polymer electrolyte. Phys. B.

[cit28] Krichen M., Chakchouk N., Elghmaz E., Hajlaoui F., Karoui K. (2025). Multifaceted spectroscopic study of electrical and optical properties in sodium manganese oxide ceramics. J. Alloys Compd..

[cit29] Gharbi I., Ghoudi A., Weslati N., Tliha M., Oueslati A. (2025). Comprehensive study of the structural, microstructural, and electrical properties of RbZnPO4: insights into conduction mechanisms and the OLPT models. Mater. Adv..

[cit30] Karoui S., Kamoun S. (2021). Study of dielectric relaxation and polaron conductivity mechanism in sodium nitroprusside (SNP):Na_2_[Fe(CN)_5_(NO)]·2H_2_O. Phys. E.

[cit31] Zaafouri A., Megdiche M. (2017). Experimental and theoretical study of AC electrical conduction mechanisms by NSPT model of LiNa3-xAgxP2O7 (x = 0.2 and 0.6) ceramic compounds. Ionics.

[cit32] Mansour Sh. A., Yahia I. S., Yakuphanoglu F. (2010). The electrical conductivity and dielectric properties of C.I. Basic Violet 10. Dyes Pigm..

[cit33] Ncib W., Ben Jazia Kharrat A., Saadi M. (2019). *et al.*, Structural, AC conductivity, conduction mechanism and dielectric properties of La_0.62_Eu_0.05_Ba_0.33_Mn_0.85_Fe_0.15_O_3_ ceramic compound. J. Mater. Sci.: Mater. Electron..

[cit34] Saidi K., Kamoun S., Ferid Ayedi H., Arous M. (2013). J. Phys. Chem. Solids.

[cit35] Rahal A., Megdiche Borchani S., Guidara K., Megdiche M. (2018). Studies of electric, dielectric, and conduction mechanism of LiNiV_0.5_P_0.5_O_4_. J. Alloys Compd..

[cit36] Nasri S. , Felhi H., Almutairi F. N., Oueslati A., Investigation into the Crystal Structure, Hirshfeld Surface Analysis, and Electrical Transport Conduction of theAg0.2Na0.8FeP2O7compound, Preprints, 2024, preprint, 10.20944/preprints202405.1404.v1

[cit37] Mendil R., Nasri S., Oueslati A. (2025). Structural investigation, vibrational study, and Na-ion transport properties of NaGaP_2_O_7_ as sodium solid electrolyte. Ionics.

[cit38] Mott N. F. and Davis Sec E. A., Electronic Processes in Non-Crystalline Materials, Clarendon Press, Oxford, 1979

[cit39] Hajlaoui S., Hajlaoui S., Amorri O. (2022). *et al.*, Structural, theoretical, and experimental study of AC electrical conduction mechanism and thermodynamic properties of Cu_0.6_Cd_0.4_Cr_2_O_4_ spinel oxide. Ionics.

